# Supplementary material for: Identification of Wheat Septoria tritici Resistance Genes in Wheat Germplasm Using Molecular Markers
Source: Plants (Basel). 2024 Apr 16;13(8):1113. doi: 10.3390/plants13081113 (PMC11054562; doi:10.3390/plants13081113)
Supplement: Supplementary file 1 [file plants-13-01113-s001.zip › plants-2885506-supplementary.pdf]

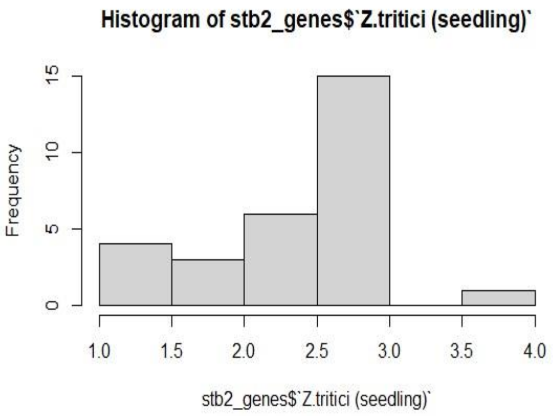

Min.	1.0
1st Qu.	2.2
Median	2.6
Mean	2.4
3rd Qu.	3.0
Max.	3.6

A

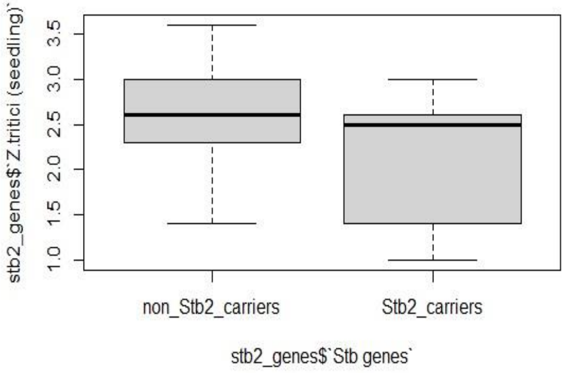

B

**Stb genes**	**Z.tritici (seedling)**
Stb2_carriers	2,2
non_Stb2_carriers	2,5
P value	0.37

Supplementary Figure S1. The general distribution of the Stb2 variable (A) and the boxplot for the Stb2 variable. (B)

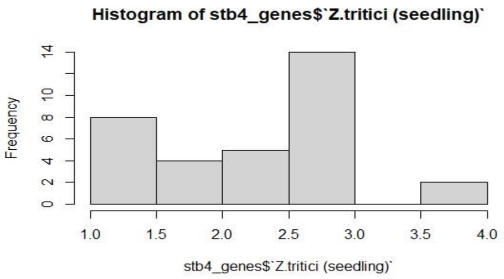

Min.	1.0
1st Qu.	1.6
Median	2.4
Mean	2.3
3rd Qu.	3.0
Max.	3.8

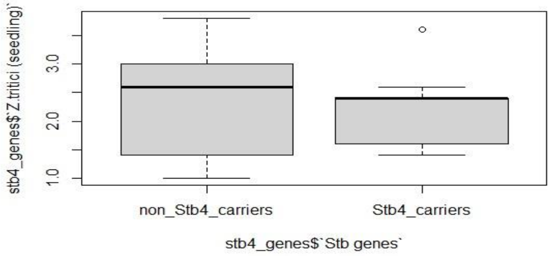

Stb genes	Z.tritici (seedling)
Stb4_carriers	2,2
non_Stb4_carriers	2,3
P value	0.51

.

A

B

Supplementary Figure S2. The general distribution of the Stb4 variable (A) and the boxplot for the Stb4 variable. (B)

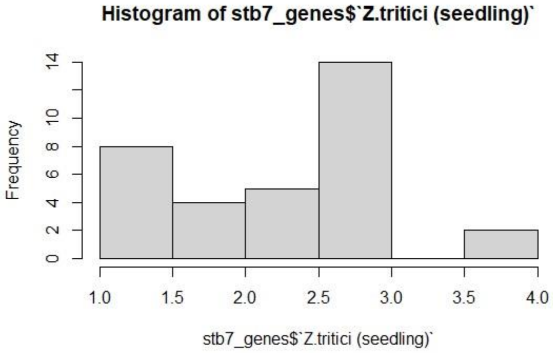

Min.	1.0
1st Qu.	1.6
Median	2.4
Mean	2.3
3rd Qu.	3.0
Max.	3.8

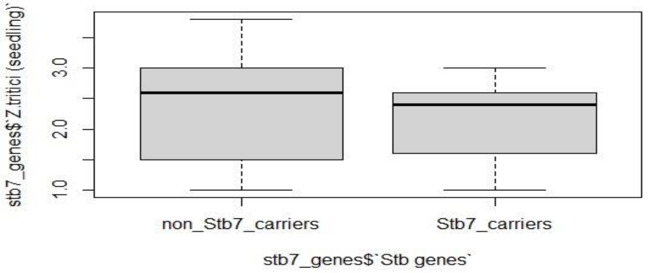

Stb genes	Z.tritici (seedling)
Stb7_carriers	2.28
non_Stb7_carriers	2,26
P value	0.62

Supplementary Figure S3. The general distribution of the Stb7 variable (A) and the boxplot for the Stb7variable (B).

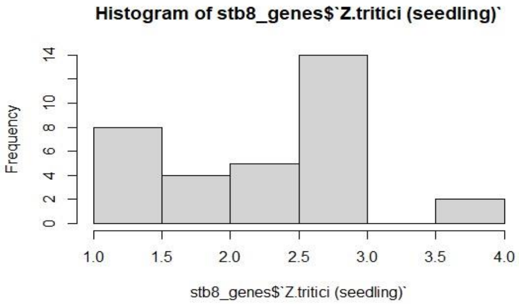

Min.	1.0
1st Qu.	1.6
Median	2.4
Mean	2.3
3rd Qu.	3.0
Max.	3.8

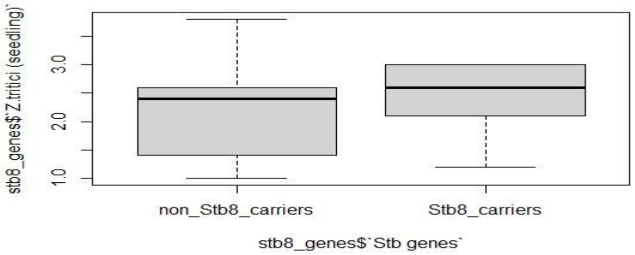

**Stb genes**	**Z.tritici (seedling)**
Stb8_carriers	2.52
non_Stb8_carriers	2.16
P value	0.19

Supplementary Figure S4. The general distribution of the Stb8 variable (A) and the boxplot for the Stb8 variable (B).

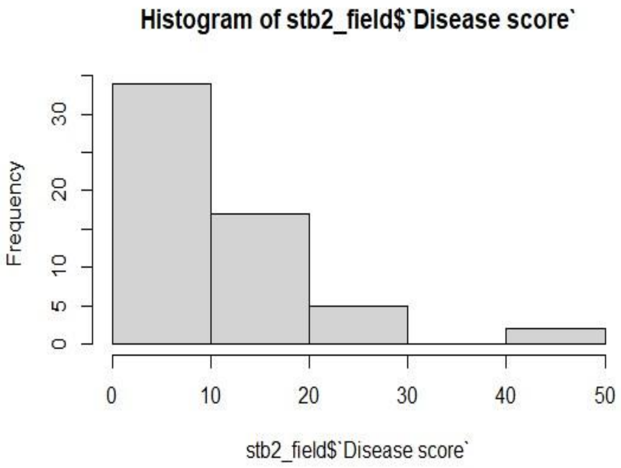

Min.	0.0
1st Qu.	6.2
Median	10.0
Mean	13.5
3rd Qu.	15.0
Max.	50.0

A

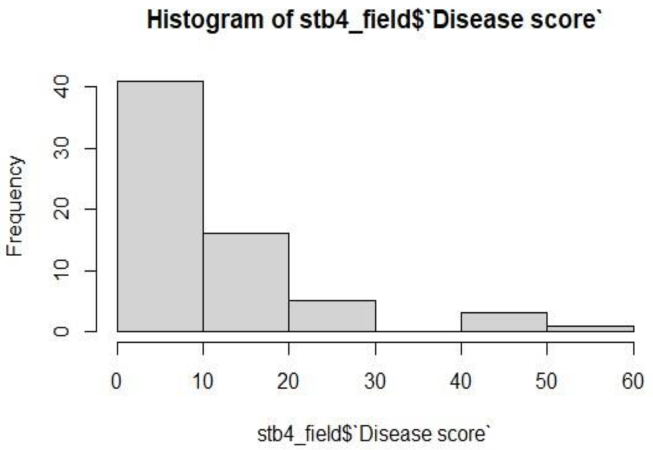

Min.	0.0
1st Qu.	5.0
Median	10.0
Mean	14.1
3rd Qu.	15.0
Max.	50.0

B

Supplementary Figure S5. General distribution of Stb2 (A) and Stb4 (B) disease scores in the field.

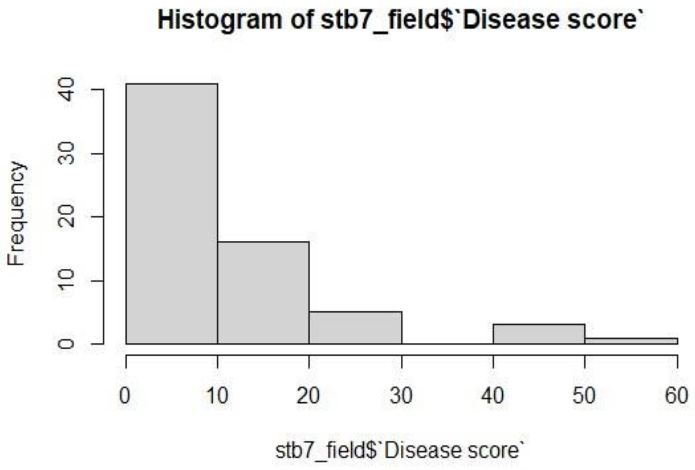

Min.	0.0
1st Qu.	5.0
Median	10.0
Mean	14.1
3rd Qu.	15.0
Max.	60.0

A

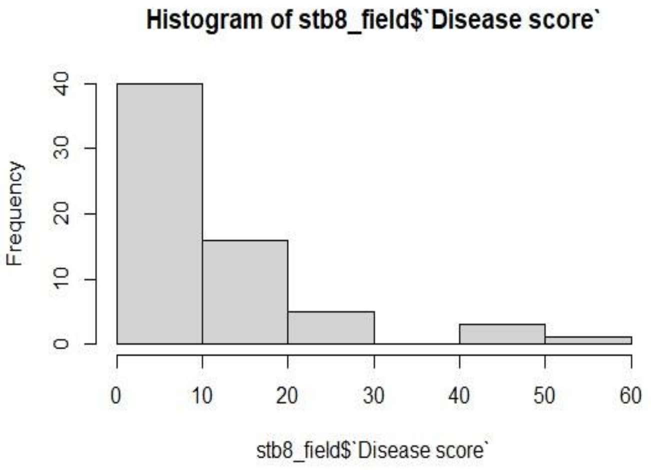

Min.	0.0
1st Qu.	5.0
Median	10.0
Mean	14.1
3rd Qu.	15.0
Max.	6.0

B

Supplementary Figure S6. General distribution of Stb7 (A) and Stb8 (B) disease scores in the field.

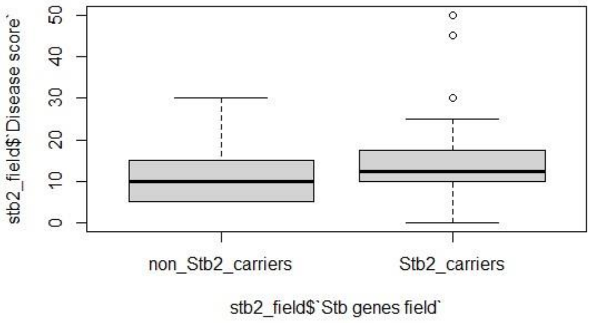

Stb genes	Z.tritici (field)
Stb2_carriers	15.7
non_Stb2_carriers	12.4
P value	0.42

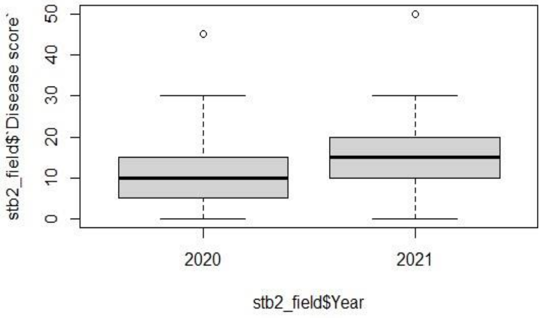

Year	Z.tritici (field)
2020	11.4
2021	15.7
P value	0.01

Supplementary Figure S7. Boxplots of Stb2 and year disease scores (2020-2021) in the field.

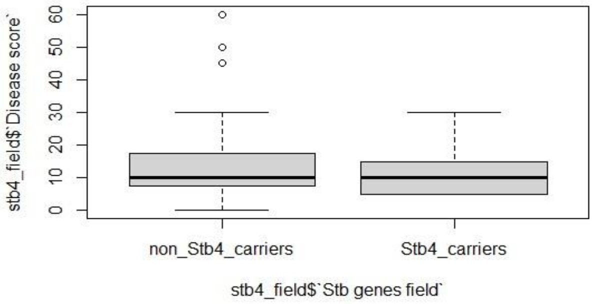

Stb genes	Z.tritici (field)
Stb4_carriers	11.1
non_Stb4_carriers	15.2
P value	0.32

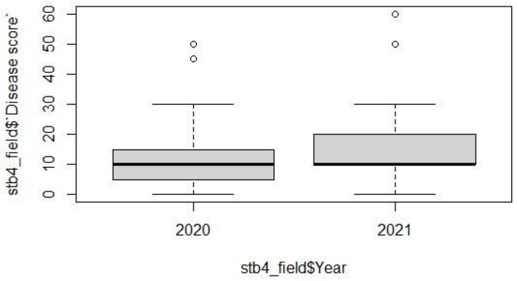

Year	Z.tritici (field)
2020	11.8
2021	16.4
P value	<0.01

Supplementary Figure S8. Boxplots of Stb4 and year disease scores (2020-2021) in the field.

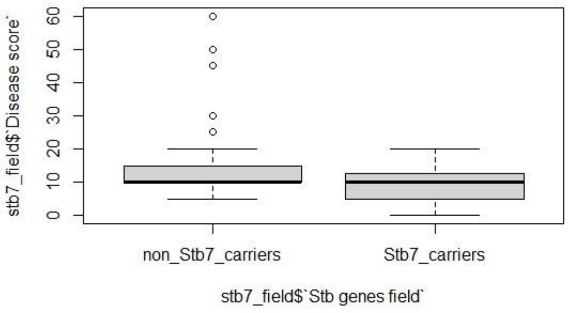

Stb genes	Z.tritici (field)
Stb7_carriers	9,2
non_Stb7_carrier s	15.2
P value	0.11

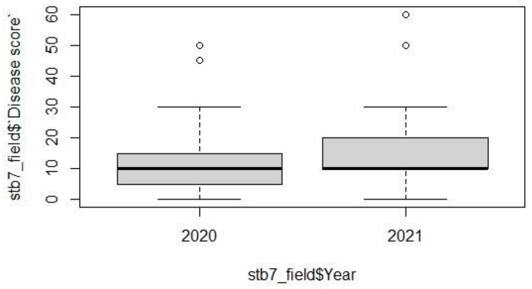

Year	Z.tritici (field)
2020	11.8
2021	16.4
P value	<0.01

Supplementary Figure S9. Boxplots of Stb7 and year disease scores (2020-2021) in the field.

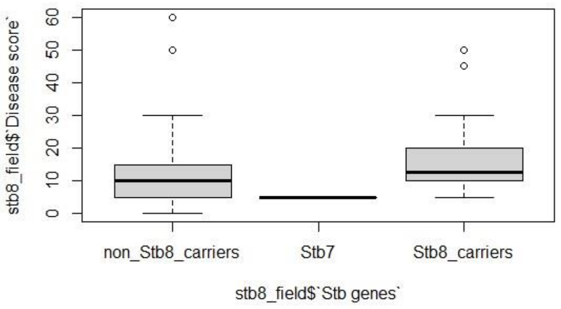

Stb genes	Z.tritici (field)
Stb8_carriers	16.1
non_Stb8_carrier s	13.1
P value	0.16

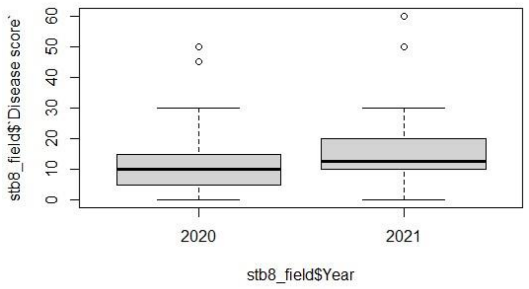

Year	Z.tritici (field)
2020	11.8
2021	16.6
P value	<0.01

Supplementary Figure S10. Boxplots of Stb8 and year disease scores (2020-2021) in the field.

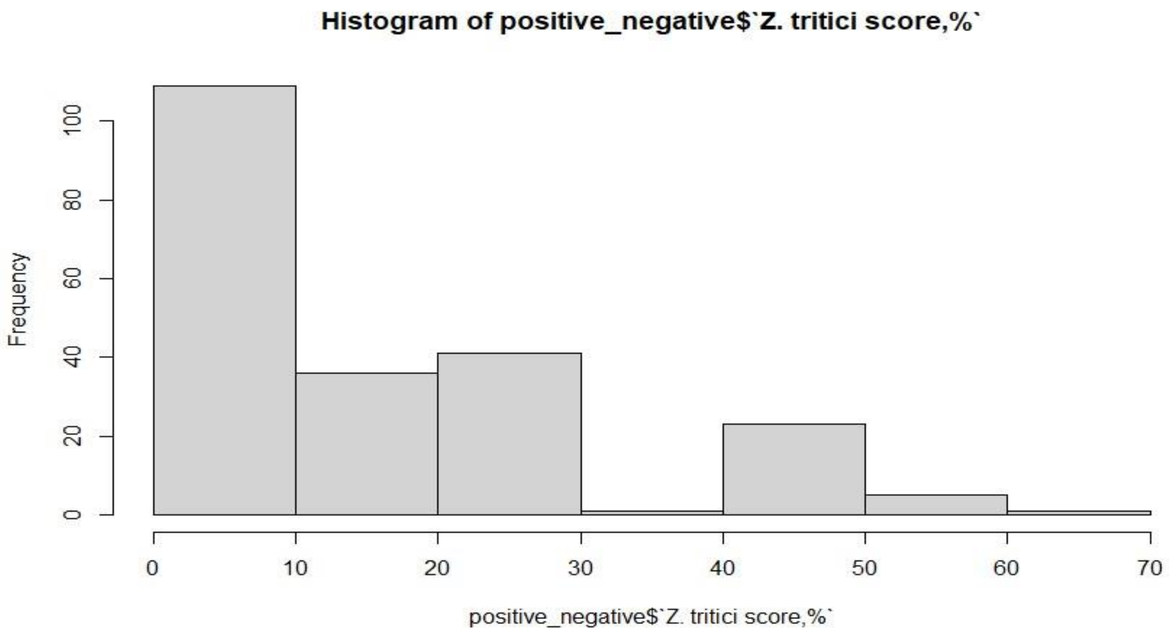

Supplementary Figure S11. The general distribution of variable severity of Z.tritici in positive and negative wheat samples concerning the presence/absence of *Stb* genes.

Min.	1st Qu	Median	Mean	3rd Qu.	Max.
0.0	10.0	10.0	18.7	25.0	65.0

**Background**	**Average *Z. tritici* score, %**
Negative	20,25
Positive	16,25
P value	<0.01

Detailed description of *Z. tritici* severity of wheat genotypes in positive (27 cvs) and negative (9 cvs) groups

Z. tritici score, %	Resistant groups	Number of Positive plants (%)	Number of Negative plants (%)
0	RR	19	0
5	RR	23	0
10	RR	22	17
15	R	6	5
20	R	5	7
25	MS	8	23
30	MS	1	1
45	S	16	47
Average disease score (P value <0.01)		20,25	16,25

Notes: According to the degree of damage to *Z. tritici*, the varieties were divided into the following groups: 0-10% – highly resistant (RR); 11-20% – resistant (R); 21-40% – moderately susceptible (MS); 41-100% – susceptible (S).
